# The mode of action of IL-23 in experimental inflammatory arthritic pain and disease

**DOI:** 10.1186/s13075-024-03380-z

**Published:** 2024-08-06

**Authors:** Kevin M.-C. Lee, Tanya Lupancu, Leon Chang, Carl L. Manthey, Martha Zeeman, Anne M. Fourie, John A. Hamilton

**Affiliations:** 1 Department of Medicine, Royal Melbourne Hospital, The University of Melbourne, Parkville, VIC 3050 Australia; 2 Janssen Research & Development, San Diego, CA USA; 3 Janssen Research & Development, Spring House, PA USA

**Keywords:** IL-23, Arthritis, Pain, Mechanism

## Abstract

**Objectives:**

We have previously reported using gene-deficient mice that the interleukin (IL)-23p19 subunit is required for the development of innate immune-driven arthritic pain and disease. We aimed to explore here, using a number of in vivo approaches, how the IL-23p19 subunit can mechanistically control arthritic pain and disease in a T- and B- lymphocyte-independent manner.

**Methods:**

We used the zymosan-induced arthritis (ZIA) model in wild-type and *Il23p19*^*−/−*^ mice, by a radiation chimera approach, and by single cell RNAseq and qPCR analyses, to identify the IL23p19-expressing and IL-23-responding cell type(s) in the inflamed joints. This model was also utilized to investigate the efficacy of IL-23p19 subunit blockade with a neutralizing monoclonal antibody (mAb). A novel IL-23-driven arthritis model was established, allowing the identification of putative downstream mediators of IL-23 in the control of pain and disease. Pain and arthritis were assessed by relative static weight distribution and histology, respectively.

**Results:**

We present evidence that (i) IL-23p19^+^ non-bone marrow-derived macrophages are required for the development of ZIA pain and disease, (ii) prophylactic and therapeutic blockade of the IL-23p19 subunit ameliorate ZIA pain and disease and (iii) systemically administered IL-23 can induce arthritic pain and disease in a manner dependent on TNF, GM-CSF, CCL17 and cyclooxygenase activity, but independently of lymphocytes, CGRP, NGF and substance P.

**Conclusions:**

The data presented should aid IL-23 targeting both in the choice of inflammatory disease to be treated and the design of clinical trials.

**Supplementary Information:**

The online version contains supplementary material available at 10.1186/s13075-024-03380-z.

## Key messages

• There are non-lymphocyte populations in joints which can produce and respond to IL-23.
• Therapeutic blockade of IL-23p19 subunit with a neutralizing monoclonal antibody can inhibit arthritic pain and disease.
• There can be an interdependence between IL-23 and the cytokines, TNF, GM-CSF and CCL17.

**Supplementary Information:**

The online version contains supplementary material available at 10.1186/s13075-024-03380-z.

## Introduction

The IL-23p19 subunit is poorly secreted by cells but, when complexed with the p40 subunit of IL-12, it forms the secreted and active cytokine, IL-23. This cytokine is often linked with helper T cell (Th17) biology with IL-17 expression being under the control of IL-23 [1]. The traditional concept of the IL-23/IL-17 axis in inflammatory diseases, such as spondyloarthritis, psoriasis and inflammatory bowel disease, is that myeloid cell-derived IL-23 engages its receptor expressed by adaptive and innate-like lymphocytes [2]. However, even though not widely reported, there is recent evidence implicating only non-lymphocyte biology in certain IL-23-dependent pathologies [3–5].

In experimental arthritis, IL-23p19 gene-deficient (*Il23p19*^*−/−*^) mice are protected from the development of collagen-induced arthritis (CIA) [6] and antigen-induced arthritis [7]. Depleting IL-23, using a neutralizing anti-IL-23p19 subunit monoclonal antibody (mAb) before disease onset, suppressed CIA severity [8, 9]; in contrast, administration of the anti-IL-23p19 subunit mAb following the first clinical signs of CIA gave no improvement. These data suggest that IL-23 is required for disease onset but not for the effector phase of arthritis. It was also reported, using the T- and B-lymphocyte-independent, monoarticular zymosan-induced arthritis (ZIA) model, that *Il23p19*^*−/−*^ mice were protected from developing arthritis pain and had reduced disease [3]. Similar data were found in an experimental autoimmune encephalomyelitis model [10]. There are clinical trial data indicating that anti-IL23p19 subunit mAb treatment met the primary endpoints and is effective in psoriatic arthritis (PsA) patients [11] but not in rheumatoid arthritis (RA) patients [12].

Not much is known about the role of IL-23 in pathological pain development [13] although pain reduction has been noted after anti-IL-23p19 subunit mAb treatment in PsA [11]. Prior work implicated TNF, GM-CSF and CCL17 in the progression of ZIA pain and disease [14, 15], while *Il23p19*^*−/−*^ mice were protected from acute T- and B-lymphocyte independent arthritis induced by recombinant TNF, GM-CSF or CCL17 [3]. There is also evidence in ZIA joints for links between IL-23 and these other cytokines at the mRNA level [3]. These findings suggest that the contribution of IL-23 to arthritis pain and disease can have links to TNF, GM-CSF and CCL17, noting that these other mediators can also themselves be induced [3, 14–17]. In neuropathic pain and skin diseases, IL-23 and other cytokines have been invoked as being part of the interactions between immune and neuronal cells driving such pain [18, 19]. There are also links between the biologies of IL-23 and neuropeptides/neurotrophins, such as nerve growth factor (NGF) [20], calcitonin gene-related peptide (CGRP) [21] and substance P [22], all of which can be important mediators in pain development in humans [23]; additionally, cyclooxygenase (COX) products, such as prostaglandin E_2_, have been linked to IL-23 biology [24, 25]. However, precisely how IL-23 contributes to arthritic pain and disease, independently of T- and B-lymphocyte involvement, requires further study.

In the current mechanistic study, using a T- and B-lymphocyte-independent arthritis model, we provide evidence for a non-bone marrow (BM)-derived resident macrophage to be the possible IL-23p19 subunit source and ILCs, fibroblasts, neutrophils and/or macrophages an IL-23-responsive cell type(s). We also report that a neutralizing IL-23p19 subunit mAb is effective in this model, even if given therapeutically, suggesting a peripheral action of IL-23 in controlling ongoing arthritic pain and disease. Also, using a new IL-23-driven arthritis model, we present data for a dependence on TNF, GM-CSF and CCL17 for pain and disease reinforcing their links with IL-23 in inflammation-associated pain and disease, as exemplified here in joints.

## Materials and methods

A detailed materials and methods section is available in the online supplemental file

### Mice

The following male mice (8–10 weeks) were used: C57BL/6 (referred to as wild-type (WT) throughout), *GM-CSF (Csf2)*^−/−^ [14], *Ccl17*^*E/E*^ [14], *Il23p19*^*−/−*^ [3] and *Rag1*^*−/−*^ mice.

### Zymosan-induced arthritis (ZIA)

ZIA was induced as published [3]. Synovial tissue cells (days 3 and 7) were subjected to flow cytometric cell sorting (see below). For monoclonal antibody (mAb) treatments, mice received i.p. injections of 150 ug of anti-IL-23p19 mAb (CNTO3723, Janssen USA) [26], anti-IL-17 A mAb (CNTO8096, Janssen USA) [27], anti-IL-1α mAb (clone ALF-161, InVivoMAb), anti-IL-1β (clone B122, InVivoMAb) mAb, CNTO6601 (Janssen USA) [28] (isotype control for anti-IL-23 and IL-17 A mAb, Janssen USA) or Armenian hamster IgG (isotype control for anti-IL-1α and anti-IL-1β mAb, InVivoMAb), either prophylactically (starting day − 1, followed by three times a week) or therapeutically (starting when pain is evident, followed by three times a week).

### Generation of bone marrow (BM) chimera

BM chimeric mice were generated as published [16].

### Behavioural pain assessment

As an indicator of pain-like behaviour (referred to as pain throughout), a ratio between two knees (left vs. right) was used as a measure of static weight-bearing joint pain using an incapacitance meter (IITC Life Science Inc, USA) and expressed as percentage weight on the contralateral hindlimb. Values between 90 and 100 for the percentage (%) weight on the contralateral hindlimb are within a normal range of variation (i.e. no pain); a value below 90 indicates pain [3, 14–16, 29].

### Flow cytometric cell sorting

As before [29], joints were digested, analyzed and sorted using BD Aria II (BD Biosciences, USA).

### Quantitative PCR (qPCR)

qPCR analysis was performed as published [3].

### Single-cell RNA-sequencing (scRNA-seq)

The viability of cells in all ZIA joint samples was assessed to be > 90%, determined using 7AAD staining (BD Biosciences). Sorted cells were labelled with sample tags using a BD™ Mouse Immune Single-Cell Multiplexing Kit (BD Biosciences, USA), counted and multiplexed, ready for single-cell capture. Single-cell capture and cDNA synthesis were performed by the BD Rhapsody Single-Cell Analysis System (BD, USA), according to the manufacturer’s recommendations. Libraries were sequenced on NovaSeq S4, performed at the Australian Genome Research Facility (AGRF) (Parkville, Victoria, Australia).

### IL-23-driven monoarticular arthritis model

The previously described methylated bovine serum albumin (mBSA)/cytokine model [3, 14–16] was adapted using exogenous IL-23 as the cytokine stimulus as follows. Monoarticular arthritis was induced by an i.a. injection of 100ug mBSA in 10ul saline into the right knee on day 0, followed by a daily subcutaneous (s.c.) injection on days 0–2 of either IL-23 (1.25ug, 2.5ug or 5ug) (Biolegend) or saline. The left knee served as a control and was injected with saline. Mice were sacrificed (day 7) for knee histological analysis. The mBSA/IL-23 arthritis model was induced in *Rag1*^*−/*−^, *GM-CSF*^*−/−*^ and *Ccl17*^*E/E*^ mice or in WT mice treated with anti-TNF mAb (clone XT22, 150ug/injection), indomethacin (1 mg/kg), CGRP_8 − 37_ (1 mg/kg), anti-NGF mAb (clone Ig30, 150ug/injection), SR-140,333 (1 mg/kg) or their controls (DMSO or isotype control) administered i.p. on days − 1, 1 and 4.

### Histopathological assessment of arthritis

At 7 days post the induction of ZIA or the mBSA/IL-23 model, histology was performed on decalcified and paraffin embedded knee joints. For ZIA, cell infiltration, bone erosion (H&E stain) and proteoglycan loss (Safranin O/fast green stain) were scored separately from 0 (normal) to 3 (severe) as before [3, 14–16]. For the mBSA/IL-23 model (H&E stain), cellular infiltration, synovitis (synovial hyperplasia), pannus formation, cartilage damage and bone erosions were scored separately from 0 (normal) to 5 (severe) as described previously [3, 14–16].

### Statistical analysis

For longitudinal incapacitance meter measurements, linear mixed effects models were used for repeated measures over time with a Dunnett post-hoc test being used when comparing the treatment groups to the control group. For histology measurements, a non-parametric Kruskal-Wallis test, following Benjamini and Hochberg adjustment for *p*-values in multiple comparison, was performed to examine differences in mean histopathologic arthritis assessments. Statistical analysis was performed using GraphPad Prism Software (10.1.0) and based on a 0.05 significance level. Data were plotted as means with corresponding standard error of the mean (SEM).

## Results

### The identification of IL-23p19-expressing and IL-23-responding cells during zymosan-induced arthritis

It has been reported by us using *Il23p19*^*−/−*^ mice that the IL-23p19 subunit is required for the development of innate immune-driven, zymosan-induced inflammatory pain as well as zymosan-induced arthritic pain and disease [3]. In the current study, to explore the mechanisms governing this IL-23p19 subunit dependence, we aimed firstly to identify IL-23p19-expressing and IL-23-responding cells that could be important for zymosan-induced arthritis (ZIA) pain and disease. ZIA is a commonly used monoarticular inflammatory arthritis model [14, 15, 30–32].

#### An IL-23p19^+^ non-bone marrow-derived cell type(s) is required for ZIA pain and maximal disease development

**Fig. 1:**
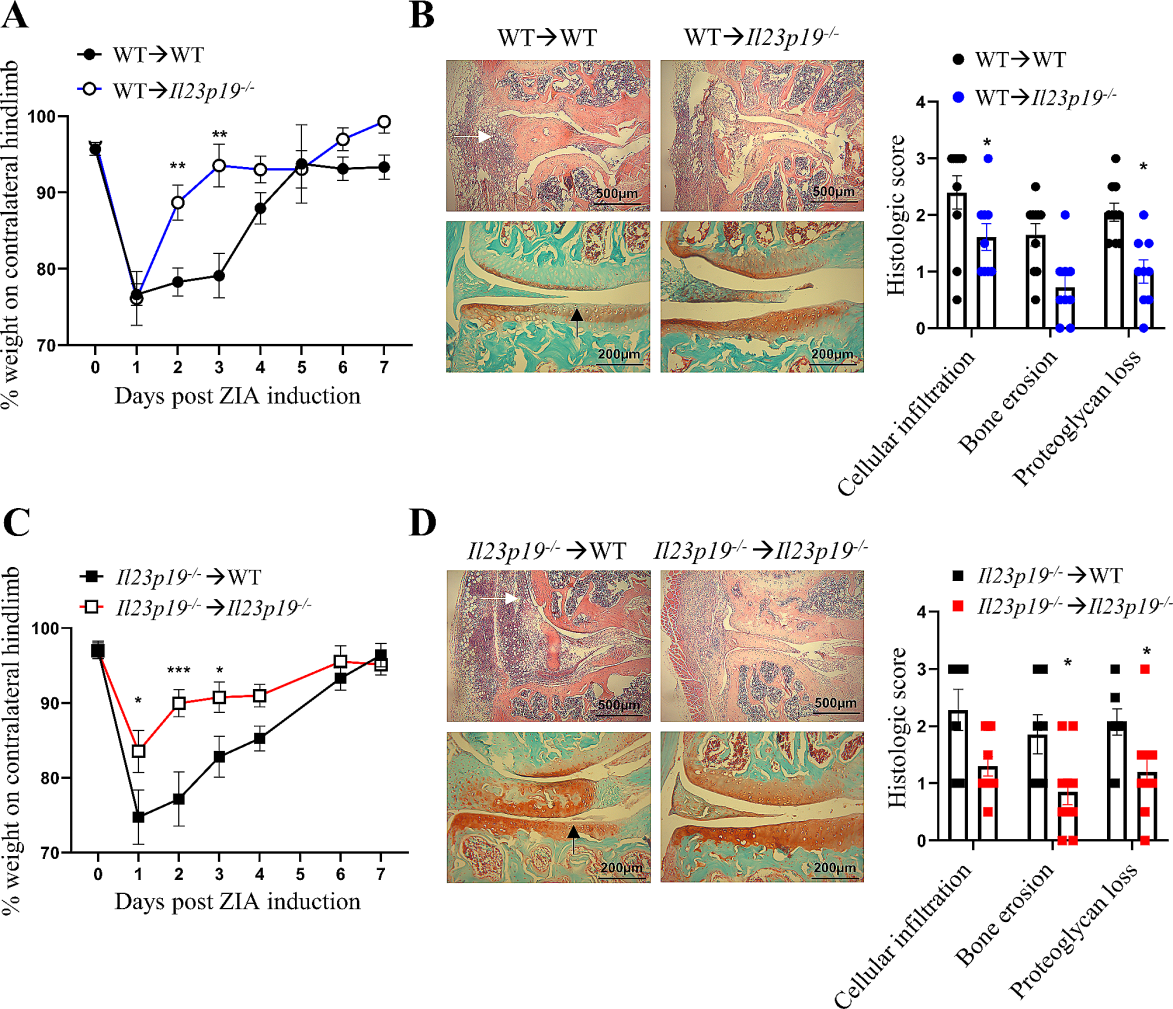
A IL23p19^+^ non-BM-derived cell(s) is required for ZIA pain and maximal disease. **A** and **B** WT or **C** and **D ***Il23p19*^*−/−*^ BM cells were adoptively transferred into irradiated WT or *Il23p19*^*−/−*^ mice, followed by the induction of the ZIA model. **A** and **C** Reduction in weight distribution (pain-like behaviour) over time. **B** and **D** Representative histologic pictures of knee joints (H&E, original magnification X40; Safranin O and Fast Green, original magnification X100) and quantification of arthritis at day 7. Data are means ± SEM (*n* = 10 mice/group). *p < 0.05, **p < 0.01, ***p < 0.001, WT→WT vs. WT→*Il23p19*^*−/−*^ mice or *Il23p19*^*−/−*^→WT vs. *Il23p19*^*−/−*^→*Il23p19*^*−/−*^ mice

Using a bone marrow (BM) chimera approach, we determined whether an *Il23p19*^*+*^ BM- or non-BM-derived cell(s) was required in the development of ZIA pain-like behaviour (referred to as pain throughout) and disease. Expectedly, the wild-type (WT) chimera (WT→WT) developed ZIA pain (Fig. 1A) and disease (Fig. 1B), whereas transfer of WT BM cells to *Il23p19*^*−/−*^ mice (WT→*Il23p19*^*−/−*^) resulted in transient pain only on day 1 (Fig. 1A) and reduced disease severity (day 7) (Fig. 1B); as expected, the *Il23p19*^*−/−*^ chimeric mice (*Il23p19*^*−/−*^→*Il23p19*^*−/−*^) did not develop pain throughout and have reduced disease severity, whereas transfer of *Il23p19*^*−/−*^ BM to WT mice (*Il23p19*^*−/−*^→WT) led to such development (Fig. 1C and D). Under the curve analysis between WT→*Il23p19*^*−/−*^ mice and *Il23p19*^*−/−*^→*Il23p19*^*−/−*^ mice showed a similar overall improvement in arthritic pain (*p* = 0.1934, 95% CI [-17.80, 3.88]). These data suggest that an *Il23p19*^*+*^ non-BM-derived cell(s) is required for ZIA pain progression and maximal disease development.

#### IL-23p19-expressing and IL-23-responding synovial cells in ZIA

**Fig. 2:**
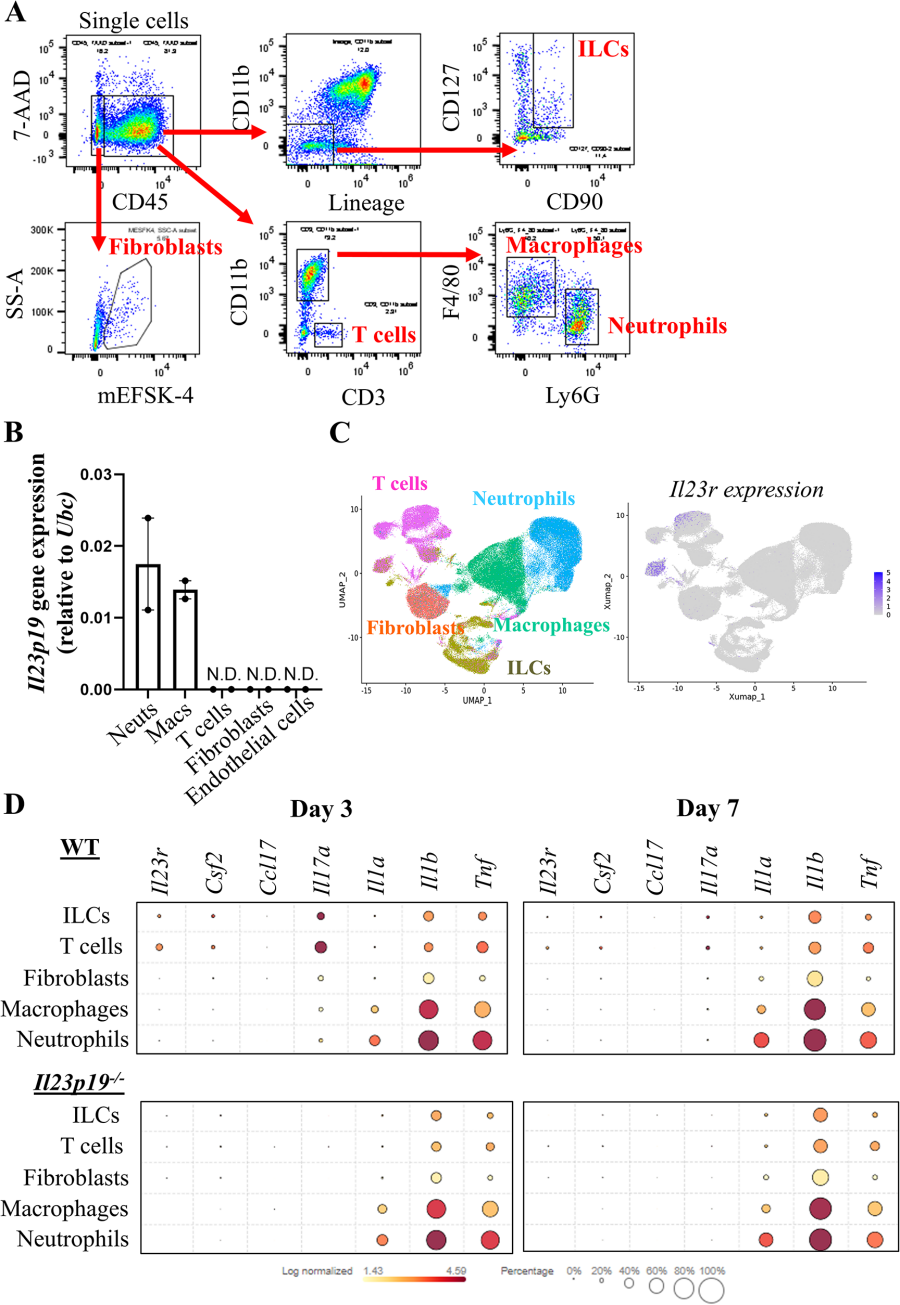
Cytokine and *Il23r* expression profiles in synovial cells during ZIA. **A** Representative gating strategy for flow cytometric sorting ZIA WT synovial tissue cells (day 7) for qPCR and scRNAseq and analysis. Lineage markers are described in Supplemental Table I. **B*** Il23p19* gene expression analysis (qPCR) on sorted ZIA WT synovial tissue cells (day 7) (*n* = 2 mice). **C** UMAP (day 7) showing the *Il23r* expression in the different ZIA WT synovial cells (colour intensity indicates average expression) (*n* = 5 mice). **D** 2D dot plot showing the expression of *Il23r*,* Csf2*,* Ccl17*,* Il17a*,* Il1a*,* Il1b* and *Tnf* in the different ZIA synovial cells (colour intensity indicates average expression) at days 3 and 7 (*n* = 5 WT, *n* = 5 *Il23p19*^*−/−*^). Data are means ± SEM. N.D. not detected

To further identify the IL-23p19-expressing cell type(s) and also to identify IL-23-responding cells during ZIA, we isolated five ZIA synovial tissue populations, namely innate lymphoid cells (ILCs), CD3^+^ lymphocytes, mESK4^+^ fibroblasts [29], F4/80^+^ macrophages and Ly6G^+^ neutrophils (Fig. 2A), and sorted them for qPCR analysis (day 7), as well as for scRNAseq analysis (days 3 and 7) using the BD Rhapsody targeted inflammation panel (see Materials and Methods).

By qPCR, we found that the *Il23p19* gene is expressed by macrophages and neutrophils but not in T cells, fibroblasts and endothelial cells during ZIA (Fig. 2B), consistent with macrophages and/or neutrophils being the source. Since the chimera data above indicate an IL-23p19^+^ non-BM-derived cell(s) is required for ZIA pain and disease, it could be that a resident (synovial) macrophage is the IL-23 source.

By scRNAseq for both days 3 and 7, the *Il23r* (Fig. 2C) gene was found predominately expressed by T cells with ILCs being the next most frequently expressed cell type (Fig. 2C); *Il23r* expression was detectable in macrophages, fibroblasts and neutrophils but was quite low (Fig. 2C).

### IL-23p19 regulation of synovial cell numbers and inflammatory cytokine gene expression in ZIA

We next examined the effect of IL-23p19 gene deletion in the ZIA model on the numbers of the sorted synovial cell populations and their expression of certain inflammatory cytokine genes.

It can be seen in Supplemental Fig. 1 that at day 7 only neutrophils showed a reduced percentage in *Il23p19*^*−/−*^ mice compared to the values in WT mice, consistent with our previous report linking IL-23 and neutrophils in zymosan-induced inflammation [33].

Inflammatory cytokines, including GM-CSF [14], CCL17 [14], IL-17 [34], IL-1α [35], IL-1β [35] and TNF [35, 36], have been implicated in the development of ZIA, some of which have also been associated with IL-23 in ZIA [3] and other pathologies [37–41]. By scRNAseq analysis, it can be observed that, at days 3 and 7, *Csf2*,* Il17a*,* Il1a*,* IL1b*, and *Tnf* expression (but not *Ccl17* expression) are found to varying degrees in the WT synovial cell populations (Fig. 2D); interestingly, both *Csf2* and *Il17a* expression (*p* < 0.05), but not that of *Il1a*, *Il1b* and *Tnf*, were lower in *Il23p19*^*−/−*^ ZIA cells compared with WT ZIA cells. The day 7 data confirmed our prior data for *Csf2* and *Tnf* in bulk synovial tissue RNA expression [3] and are consistent with the literature linking IL-23 biology with IL-17 A [37–40] and GM-CSF [38, 40, 41]. Interestingly, *Il23p19*^*−/−*^ mice showed lower expression of the *Il23r* gene across the different populations (*p* < 0.05) indicating some IL-23p19 dependence (Fig. 2D).

### IL-23p19 blockade ameliorates ZIA pain and arthritis

The evidence for the IL-23p19 subunit requirement for ZIA pain and disease development was obtained so far using only gene-deficient mice [3]. To confirm the requirement of endogenous IL-23p19 subunit and to explore further its mode of action during ZIA, we utilized a timed neutralizing mAb approach. We also included neutralizing mAbs to IL-17 A, IL-1α and IL-1β by way of comparison.

#### Prophylactic blockade

**Fig. 3:**
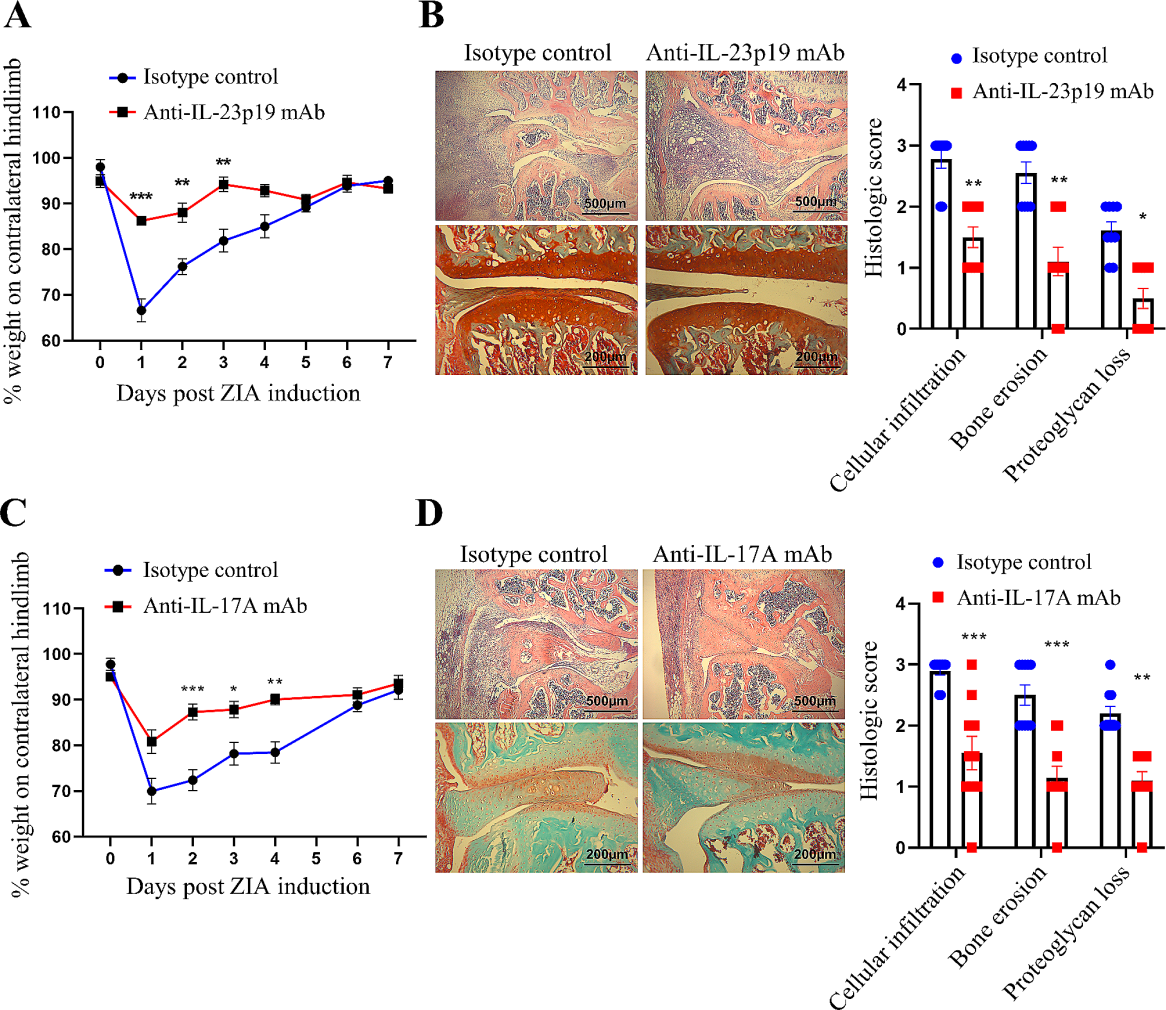
IL-23 and IL-17 A are required for the development of ZIA pain and maximal disease. **A** and **B** anti-IL-23p19 subunit mAb (150 µg) or **C** and **D** anti-IL-17 A mAb (150 µg) was administered prophylactically on day − 1, 1 and 4 in WT mice with day 0 being the induction of the ZIA model. **A** and **C** Reduction in weight distribution (pain-like behaviour) over time. **B** and **D** Representative histologic pictures of knee joints (H&E, original magnification X40; Safranin O and Fast Green, original magnification X100) and quantification of arthritis at day 7. Data are means ± SEM (*n* = 10 mice/group). *p < 0.05, **p < 0.01, ***p < 0.001, isotype control vs. anti-IL-23p19 subunit mAb or isotype control vs. anti-IL-17 A mAb

We administered the mAbs and the respective isotype controls on days − 1, 1 and 4 to WT mice induced with ZIA on day 0 (prophylactic protocol). Blocking IL-23p19 subunit or IL-17 A effectively prevented the development of ZIA pain (Fig. 3A and C) and reduced disease severity (Fig. 3B and D). While anti-IL-1α mAb had an no effect on ZIA pain (Supplemental Fig. 2A) or disease development (Supplemental Fig. 2B), anti-IL-1β mAb showed a trend towards improvement in the degree of ZIA pain (Supplemental Fig. 2C), but not in disease development (Supplemental Fig. 2D).

#### Therapeutic blockade

**Fig. 4:**
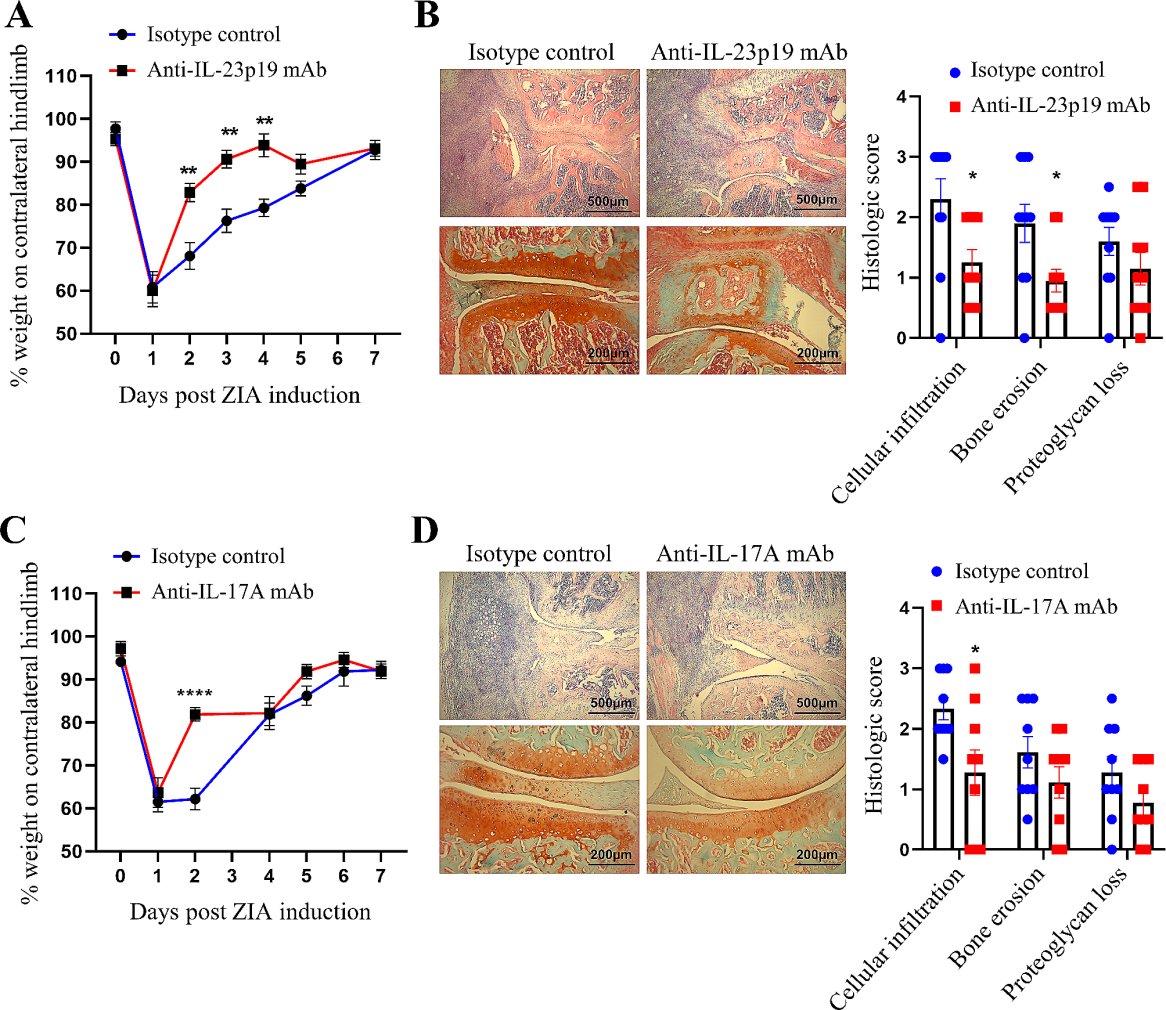
IL-23 and IL-17 A are required for the progression of ZIA pain and disease. **A** and **B** anti-IL-23p19 subunit mAb (150 µg) or **C** and **D** anti-IL-17 A mAb (150 µg) was administered therapeutically on day 1 and 4 in WT mice with day 0 being the induction of the ZIA model. **A** and **C** Reduction in weight distribution (pain-like behaviour) over time. **B** and **D** Representative histologic pictures of knee joints (H&E, original magnification X40; Safranin O and Fast Green, original magnification X100) and quantification of arthritis at day 7. Data are means ± SEM (*n* = 10 mice/group). *p < 0.05, **p < 0.01, ****p < 0.0001, isotype control vs. anti-IL-23p19 subunit mAb or isotype control vs. anti-IL-17 A mAb

We continued testing the efficacy of anti-IL-23p19 subunit and IL-17 A mAbs by administering them on days 1 and 4 (therapeutic protocol). Following ZIA induction, anti-IL-23p19 subunit mAb administration rapidly and effectively ameliorated already established arthritic pain (Fig. 4A) and reduced disease severity (Fig. 4B); anti-IL-17 A mAb also ameliorated established arthritic pain and prevented somewhat disease progression (Fig. 4C and D).

These data indicate that the IL-23p19 subunit and IL-17 A are important throughout the course of the innate immune-driven ZIA pain and disease.

### IL-23-driven arthritic pain and disease

#### An IL-23-driven arthritis model

**Fig. 5:**
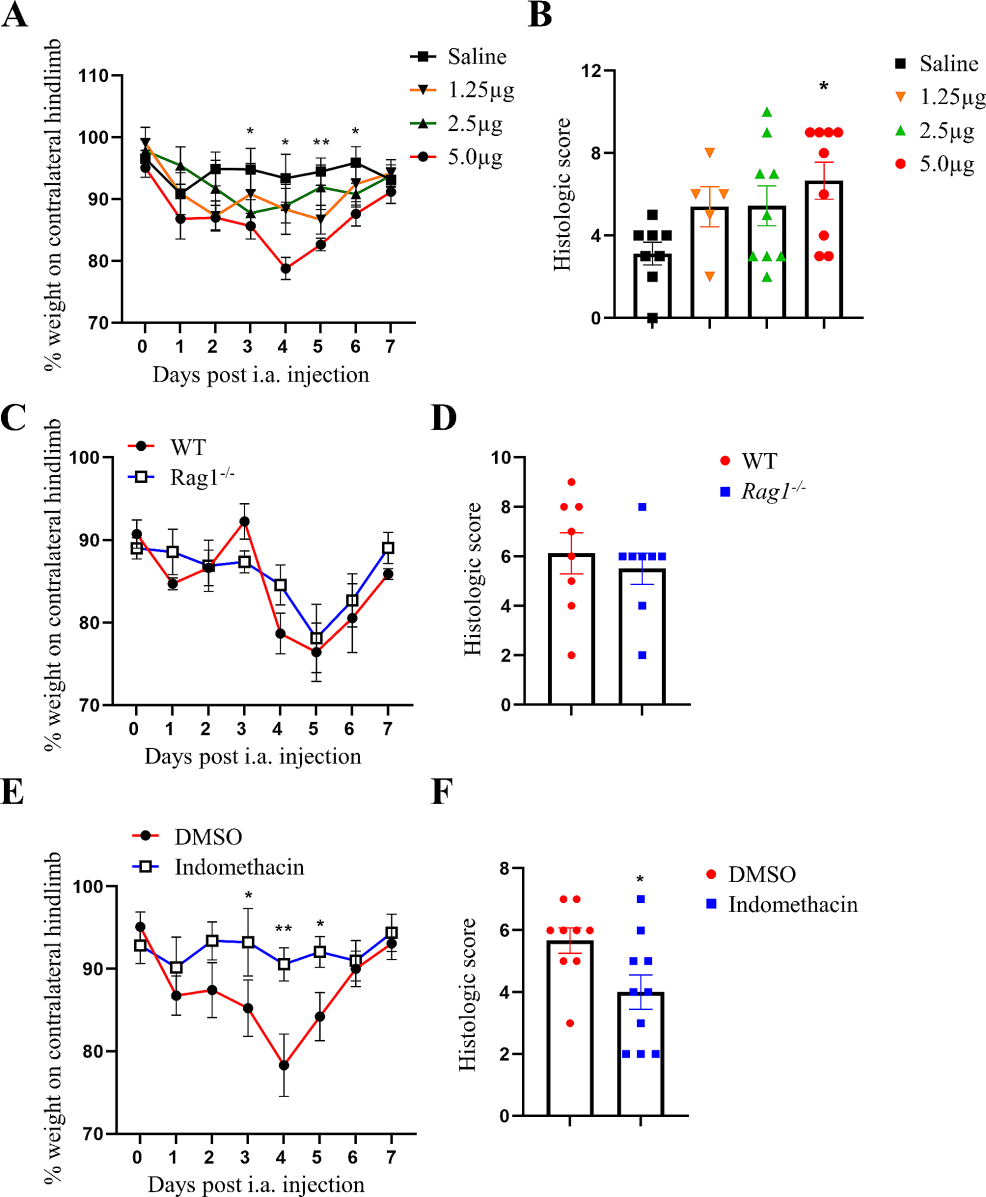
IL-23-driven arthritic pain and disease do not require T- and B-lymphocytes but do require cyclooxygenase activity. **A** and **B** Optimization of IL-23-driven arthritic pain and disease (i.a. mBSA [day 0] and s.c. IL-23 [1.25 µg, 2.5–5 µg] or saline (days 0 to 2) in WT mice. mBSA/IL-23 arthritis (i.a. mBSA [day 0] and s.c. IL-23 [5 µg]) was induced in (**C** and **D**) WT or *Rag1*^*−/−*^ mice or (**E** and **F**) in WT mice treated with either DMSO (control) or indomethacin (1 mg/kg; days − 1, 1 and 4). **A**, **C** and **E** Reduction in weight distribution (pain-like behaviour) over time. **B**, **D** and **F** Quantification of arthritis at day 7. Data are means ± SEM (*n* = 5–10 mice/group). *p < 0.05, **p < 0.01, 5 µg IL-23 vs. saline or DMSO vs. indomethacin

We next adapted the so-call methylated bovine serum albumin (mBSA)/cytokine monoarticular arthritis model [3, 14–16] with exogenous IL-23 to determine whether we could develop an IL-23-driven arthritis model to explore putative downstream pathways. WT mice received an i.a. injection of mBSA on day 0 and increasing doses of s.c. IL-23, or saline as control, on days 0–2. IL-23 administered at a dose of 5 µg, but not 1.25 and 2.5 µg, induced significant arthritic pain on day 3 (*p* = 0.0008, 5 µg vs. saline) (Fig. 5A) and disease on day 7 (*p* = 0.036, 5 µg vs. saline) (Fig. 5B). Subsequent experiments utilized 5 µg IL-23 in this new arthritis model.

#### The role of T- and B-lymphocytes and COX activity in IL-23-driven arthritis

As mentioned, IL-23 is often intimately linked with T cell biology [1, 42]; also IL-23-driven inflammatory pain requires COX activity [3]. We thus analyzed the T- and B-lymphocyte dependence of pain and disease in the mBSA/IL-23 arthritis model using *Rag1*^*−/−*^ mice and a COX inhibitor.

For the mBSA/IL-23 arthritis model in *Rag1*^*−/−*^ mice, we found a similar degree of pain (Fig. 5C) and arthritis (Fig. 5D) development as for WT mice, suggesting mature T- and B-lymphocyte independence. Following administration of the COX inhibitor, indomethacin, IL-23-driven arthritic pain was prevented (Fig. 5E), which is consistent with our previous findings using an IL-23-driven inflammatory pain model [3]; interestingly, indomethacin also inhibited somewhat the progression of IL-23-driven arthritis (Fig. 5F). These data suggest that IL-23-driven arthritic pain and histologic disease require COX activity but not T- and B-lymphocytes.

#### The role of TNF, GM-CSF and CCL17 in IL-23-driven arthritis

We have reported previously, using *Il23p19*^*−/−*^ mice, that IL-23 is required for TNF-, GM-CSF- and CCL17-driven arthritic pain and disease development [3], indicating possible links between IL-23 and these cytokine mediators, which in turn have themselves been linked in the mBSA/cytokine arthritis models [3, 14, 15] and elsewhere in other models [14]. We therefore examined the downstream dependence of IL-23 on TNF, GM-CSF and CCL17, using the mBSA/IL-23 model with a neutralizing mAb approach or gene-deficient mice.

**Fig. 6:**
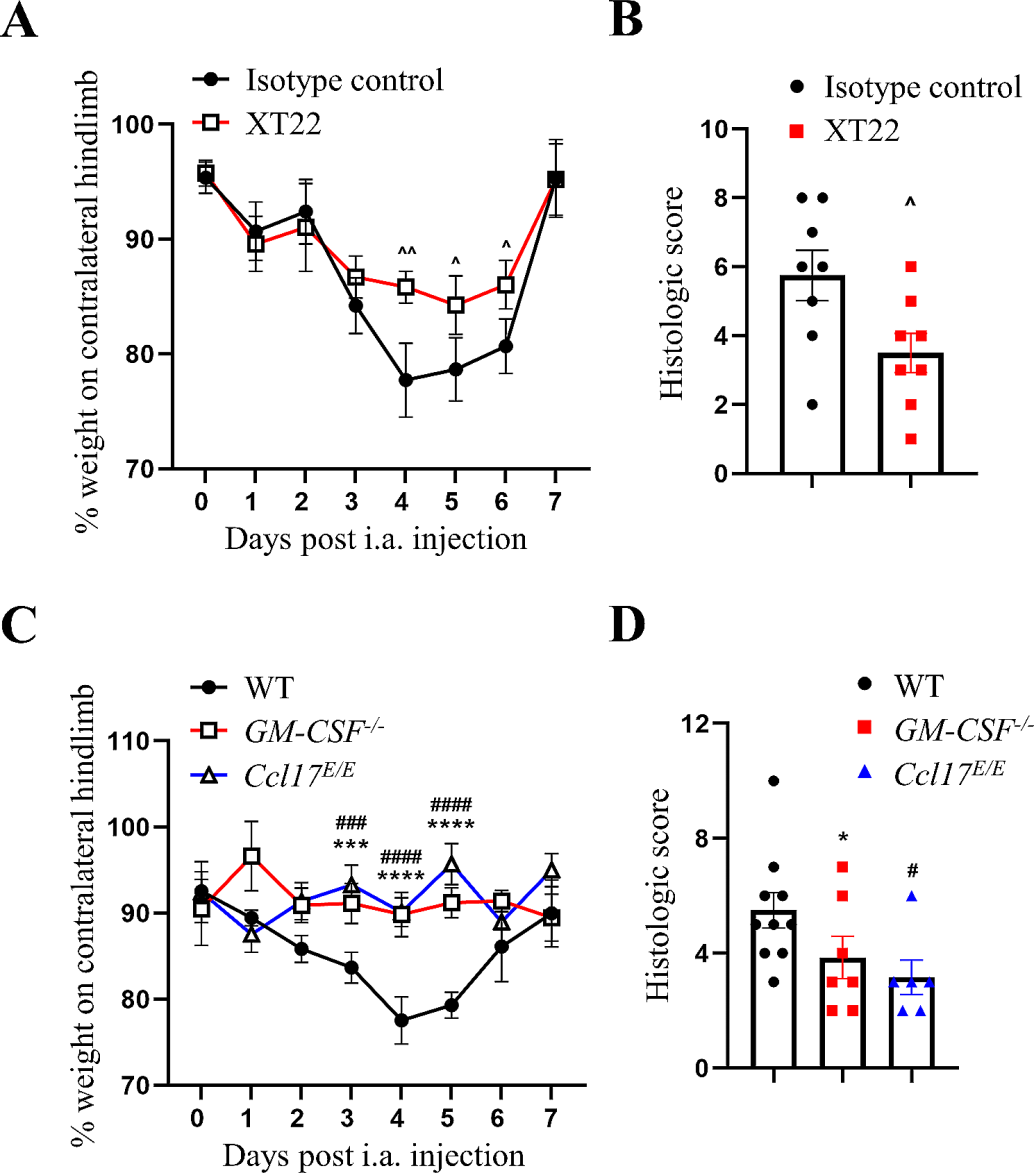
IL-23-driven arthritic pain and maximal disease require TNF, GM-CSF and CCL17. mBSA/IL-23 arthritis (i.a. mBSA [day 0] and s.c. IL-23 [5 µg]) was induced (**A** and **B**) in WT mice treated with either isotype control or anti-TNF mAb (clone XT22; 150 µg; on days − 1, 1 and 4) and (**C** and **D**) in WT, GM-CSF^−/−^ and *Ccl17*^*E/E*^ mice. (**A** and **C**) Reduction in weight distribution (pain-like behaviour) over time. (**B** and **D**) Quantification of arthritis at day 7. Data are means ± SEM (*n* = 8–10 mice/group). ^*p* < 0.05, ^^*p* < 0.01, isotype control vs. XT22; *p < 0.05, ***p < 0.001, ****p < 0.0001, WT vs. *GM-CSF*^*−/−*^; #*p* < 0.05, ###*p* < 0.001, ####*p* < 0.0001, WT vs. *Ccl17*^*E/E*^

The administration of an anti-TNF mAb prevented the development of IL-23-driven arthritic pain (Fig. 6A) and reduced arthritis severity (Fig. 6B). In addition, IL-23-driven arthritic pain (Fig. 6C) was not seen in *GM-CSF*^*−/−*^ and *Ccl17*^*E/E*^ mice, while reduced arthritis severity (Fig. 6D) was observed. These data indicate that IL-23-driven arthritic pain and maximal disease are also dependent on TNF, GM-CSF and CCL17.

#### The role of NGF, CGRP and substance P in IL-23-driven arthritis

Given the data above showing COX activity involvement in IL-23-driven arthritic pain and disease, we explored if other important mediators in pain development, namely nerve growth factor (NGF), calcitonin gene related peptide (CGRP) and substance P, are required. For this purpose, we used a neutralizing anti-NGF mAb and antagonists of CGRP (CGRP_8 − 37_ peptide) or substance P (SR140333), as previously described [16]. None of the inhibitors suppressed mBSA/IL-23 arthritic pain and disease development (Supplemental Fig. 3A-F).

## Discussion

We explored above how IL-23 can control arthritic pain and disease, the former readout not widely studied in relation to the IL-23 pathway. The focus in this study was on T- and B-lymphocyte-independent arthritis models, systems also not usually studied for IL-23 biology.

In the ZIA model, evidence was provided, using a chimera approach and qPCR analysis, that non-BM-derived synovial tissue macrophages could be a source of IL-23p19 and represent a key to pain and disease development. The reduced neutrophil percentage in the ZIA joints of *Il23p19*^*−/−*^ mice is consistent with our recent proposal during zymosan-induced peritonitis that IL-23p19 regulates the neutrophilic response via G-CSF [33]. Consistent with this proposal, we have published previously that mice subject to anti-GCSFR or anti-Ly6G (i.e. neutrophil depletion) blockade are protected from ZIA development [43]. Other studies have also implicated IL-23 being involved in granulopoiesis via an IL-17/G-CSF pathway [33, 44, 45].

The scRNAseq analysis of cytokine gene expression in ZIA synovial tissue cell populations (Fig. 2D) supports links also between IL-23 biology and those of TNF, GM-CSF and IL-17, as suggested before [37–40]. In the same analysis it appears that the *Il23r* gene is expressed across all the populations studied, namely T-lymphocytes and ILCs, with low expression being found in macrophages, fibroblasts and neutrophils. The T- and B-lymphocyte-independence of the ZIA model [14, 15] leaves the ILCs, macrophages, fibroblasts and neutrophils as potentially relevant IL-23-responding cell populations [5, 33, 46–49]. Additional studies are required as only limited cell types were sorted for scRNAseq analysis. The apparent IL-23p19-dependence of *Il23r* gene expression could help explain any reduced inflammatory mediator gene expression observed in the *Il23p19*^*−/−*^ ZIA synovial cells at the single cell level (Fig. 2D).

We also found that an anti-IL-23p19 subunit mAb successfully suppressed ZIA pain and disease, both prophylactically and therapeutically; the rapid suppression by the latter protocol is particularly striking as in experimental inflammation models only prophylactic administration of anti-IL-23p19 subunit mAb is usually successful [8, 9]. In its control of arthritic pain, IL-23 can potentially be acting peripherally and/or centrally; as discussed before [3, 7, 50, 51], the effectiveness of the neutralizing mAb suggests a peripheral mechanism at least in the ZIA model. The effectiveness of the anti-IL-17 A mAb in the ZIA model points again towards a link with IL-23 [1, 42]. Further studies using the neutralizing anti-IL-23p19 subunit mAb approach in the ZIA model could be undertaken to confirm the mechanistic data obtained above in the *Il23p19*^*−/−*^ mice. From our studies IL-1α or IL-1β appear not to be important in the ZIA model even though they are both highly expressed at the gene level in the ZIA synovial cells (Supplemental Fig. 2). It should also be noted that, although the measurement of changes in static weight bearing as an indicator of arthritic pain is a relevant and well-established technique [14, 16, 29, 36, 43, 52–54], we acknowledge that it does not fully capture the pain phenotype and other measurements of pain-like behaviour should be examined.

Along with TNF and IL-23 [3, 15], we have reported that GM-CSF and CCL17 are also required for ZIA pain and histologic disease development and COX2 for pain development [14]. These data indicate a correlation between these mediators in the ZIA model and even possible links. To explore further the possible mechanistic links, we utilized the monoarticular mBSA/cytokine arthritis model [3, 14–16]. Using this approach and IL-23p19 subunit blockade/deletion strategies with TNF, GM-CSF or CCL17 as the driving stimulus, we suggested before that the IL-23p19 subunit could somehow lie downstream of each of these cytokines in controlling arthritic pain and disease [3]; we also found a COX2 activity requirement for pain progression. In the same study [3], we also found that intraplantar IL-23 could induce inflammatory, COX-dependent pain in WT mice which was not found in *Tnf*-, *Csf2*- and *Ccl17*-gene deficient mice suggesting that conversely these cytokines might lie downstream of IL-23 in the regulation of pain. These findings indicate that there might be feedback “loops” governing this group of interdependent cytokines in certain inflammatory conditions, including those where pain is a significant outcome. We therefore tested whether we could establish an IL-23-driven arthritis model that could assist in determining putative downstream mediators of IL-23 action.

We found here that systemically administered IL-23 could elicit pain and arthritis in a mBSA-injected joint and that neutralization of TNF and deletion of *Csf2* and *Ccl17* prevented the pain and reduced the associated arthritis. It was also found that the pain, and interestingly, maximal arthritis development requires COX activity. This new IL-23-driven arthritis model may prove useful in defining additional mediators/pathways downstream of the algesic and arthritogenic action of IL-23. However, this model is driven by systemic cytokine administration and is a two-stage model – the mBSA “priming” may contribute to the subsequent cytokine effects [3, 14–16]. It is interesting that intraplantar IL-23-induced pain [3] is dependent on these same mediators as for IL-23-driven arthritic pain in our new mBSA/IL-23 arthritis model (Fig. 5). It is perhaps worth noting that in two T- and B-lymphocyte-independent arthritis models in which IL-23 is induced or systemically administered there appears to be an association with the same cytokines, namely, TNF, GM-CSF and CCL17, in controlling pain and disease. Further studies are needed to understand when and how IL-23 and the other cytokines exhibit such reciprocal interdependence. The existence of such interdependence may not be at all that surprising given the prior evidence for the links, for example, between TNF and IL-23 [3], TNF and GM-CSF [15], GM-CSF and IL-23 [17, 41], and the evidence of a GM-CSF/CCL17 axis in monocytes/macrophages [14] and in RA patients [55, 56].

It is intriguing that NGF, CGRP and substance P did not seem to be required for the IL-23-driven arthritic pain and disease given that these neurotrophins/neuropeptides have been linked to the biology of IL-23 and the other cytokines discussed above [18, 20–22, 57, 58]. IL-23 expression can be detected in dorsal root ganglia [19, 59]; it would be of interest to explore how IL-23 can contribute to the activation of nociceptors for pain development and the contribution of the interdependent cytokines discussed above and of COX metabolites to this development [50, 51].

IL-23 is often associated with Th17 biology via the IL-23/IL-17 axis [1, 42]; however, as mentioned, there is some recent evidence for IL-23 to be involved in non-lymphocyte biology [3–5]. We present data here from mechanistic studies using T- and B-lymphocyte-independent arthritis models, indicating (i) the IL-23p19 subunit cellular source and responsive cell type, (ii) further evidence in inflammation for a possible interdependence between IL-23 and other cytokines, namely TNF, GM-CSF and CCL17 and (iii) importantly, the successful therapeutic blockade of arthritic pain and disease with a neutralizing anti-IL-23p19 subunit mAb consistent with a peripheral action of IL-23 in the control of ongoing pain and disease. Given that our studies above were with only two models, further studies are required to assess how general our findings and conclusions are about how IL-23 might be acting during inflammatory responses. The data presented above should aid IL-23 targeting both in the choice of inflammatory disease to be treated and the design of clinical trials.

### Electronic supplementary material

Below is the link to the electronic supplementary material.

Supplementary Material 1

## Data Availability

No datasets were generated or analysed during the current study.
