# Supplementary material for: The mode of action of IL-23 in experimental inflammatory arthritic pain and disease
Source: Arthritis Res Ther. 2024 Aug 6;26:148. doi: 10.1186/s13075-024-03380-z (PMC11302168; doi:10.1186/s13075-024-03380-z)
Supplement: Supplementary file 1 — Supplementary Material 1 [file 13075_2024_3380_MOESM1_ESM.docx]

**Supplemental Materials and Methods**

**Mice**

The following male mice (8-10 weeks) were used: C57BL/6 (referred to as wild-type (WT) throughout), *GM-CSF (Csf2)*^-/-^ (Ludwig Institute for Cancer Research) (1), *Ccl17^E/E^* (in which both copies of *Ccl17* have been replaced by enhanced green fluorescent protein (EGFP)) (1), *Il23p19^-/-^* (2) and *Rag1^-/-^* (purchased from Walter and Eliza Hall Institute of Medical Research). All gene-deficient mice were backcrossed onto the C57BL/6 background for more than 10 generations. All mice (n=2-5 mice/cage) were kept under a 12-hour light and dark cycle at 22°C and allowed free access to food and water *ad libitum.*

**Generation of bone marrow (BM) chimera**

As before (3), WT or *Il23p19^-/-^* recipient mice received total body irradiation (two exposures x 5.5 Gy, 3h apart). BM cells were harvested from the femurs and tibia of WT and *Il23p19^-/-^* donor mice and 5x10^6^ cells injected i.v. into the irradiated recipient mice. Effective BM reconstitution was determined 6 weeks later by flow cytometry using mAbs against different congenic CD45 allotypes [CD45.1 and CD45.2] (see Supplemental Table I) where possible; only Ly5.2 *Il23p19^-/-^* mice were available. It was determined that the reconstitution rate was approximately 95% (**data not shown**).

**Behavioural pain assessment**

Static weight distribution was measured with an incapacitance meter (IITC Life Science Inc) as previously reported (1-6). Researchers were blinded to mouse treatments and genotypes. As an indicator of pain-like behaviour (referred to as pain throughout), a ratio between the two knees (left vs. right) was used as a measure of static weight-bearing joint pain and expressed as percentage weight on the ipsilateral hindlimb. A reduction in weight distribution indicates pain. This technique has been validated for pain measurements in many arthritis models (1-6). Mice were acclimatized to the incapacitance meter on at least three occasions prior to the commencement of the experiment. Three measurements were taken for each time point and averaged. All equipment was pre-calibrated. A lower ratio resulting from decreased weight bearing on the operated knee indicates increased pain.

**Flow cytometry and cell sorting**

Joints were digested, as before (5) using RPMI supplemented with type II collagenase (1mg/ml), neutral proteases (0.5mg/ml) and DNAse (100ug/sample) at 37°C for 30 mins. Single cell suspensions were prepared and stained for flow cytometry analysis as previously (5). Briefly, Fc receptors on cells were blocked with normal mouse serum (1/4 dilution) on ice for 20 mins and stained on ice for 20 mins with fluorochrome-conjugated mAbs and the corresponding isotype controls (BD Biosciences, Biolegend, eBiosciences or Miltenyi Biotec) (see Supplemental Table I). Cells were analyzed and sorted using BD Aria II (BD Biosciences, USA).

**Quantitative PCR (qPCR)**

Briefly, total RNA from sorted ZIA cells was extracted using Isolate II RNA Mini Kit (Bioline, Taunton, MA) and reverse transcribed using SuperScript III reverse transcriptase (Life Technologies). qPCR was carried out using QuantStudio^TM^ 5 (Applied Biosystems^TM^, Carlsbad, CA) and pre-developed TaqMan probe/primer combinations for mouse (*Il23p19* and *Ubc*) (Life Technologies). All samples were assayed in duplicate. Threshold cycle numbers were transformed to ΔCt values, and the results were expressed relative to the reference gene, *Ubc* (7)*.*

**Single-cell RNA-sequencing (scRNA-seq)**

The FASTQ sequencing files were uploaded to the BD Rhapsody Targeted Analysis Pipeline (Version 1.10) within SevenBridges, according to the manufacturer’s directions. Alignment was performed using the Immune Response Target Panel for Mouse, which detects 397 genes, and the Single-Cell Multiplexing Kit, which allows for combining the five labeled cell types into one sequencing run. The pipeline first filters by read quality, then annotates R1 reads including the unique molecular identified (UMI), then filters validate R2 reads. R1 and R2 reads with the same cell label, same UMI and the same bioproduct are collapsed into a single raw molecule, and the reads per raw molecule are reported as the raw adjusted sequencing depth. The reads were then corrected using recursive substitution error correction (RSEC). The five sample tags were used to remove multiplets or undetermined cells, and assigned cell type labels. After this quality control and data processing, the resulting dataset includes 167,584 high quality, single cells.

**Statistical analysis**

For longitudinal incapacitance meter measurements, linear mixed effects models were fitted with unstructured covariance structure to account for repeated measures over time. Fixed-effect terms included indicators for group (isotype control or mAbs, DMSO or inhibitors, and genotypes), time in days, and their interaction. A Dunnett post-hoc test was used when comparing the treatment groups to the respective control groups. Model assumption of normality of residuals was checked visually with a Q-Q plot; equal variance of residuals was checked with a plot of residual vs fitted values.

For histology measurements, Shapiro-Wilk and Levene’s tests were used for assessing normality of data and homogeneity of variance. Due to the violation of normality assumption, which did not improve after the logarithmic or square root transformation, a non-parametric Kruskal-Wallis test, following Benjamini and Hochberg adjustment for *p*-values in multiple comparison, was performed to examine differences in mean histopathologic arthritis assessments.

Statistical analysis was performed using GraphPad Prism Software (10.1.0) and based on a 0.05 significance level. Plots were generated using GraphPad Prism Software (10.1.0). Data were plotted as means with corresponding standard error of the mean (SEM).

**Supplemental Table**

**Supplemental Table I. Flow cytometry antibodies**

| Antigen | Clone |
| --- | --- |
| CD45.1 | A20 |
| CD45.2 | 104 |
| CD45 | 30-F11 |
| mEFSK-4 | mEFSK-4 |
| CD11b | M1/70 |
| CD3 (lineage) | 145-2C11 |
| F4/80 | BM8 |
| Ly6G | 1A8 |
| CD115 (lineage) | AFS98 |
| MHCII (lineage) | M5/114.1.2 |
| CD11c (lineage) | HL3 |
| CD5 (lineage) | 53-7.3 |
| TER119 (lineage) | TER-119 |
| CD19 (lineage) | 1D3 |
| CD90.2 | 53-2.1 |
| CD127 | SB/199 |

**Supplemental Figures**

**
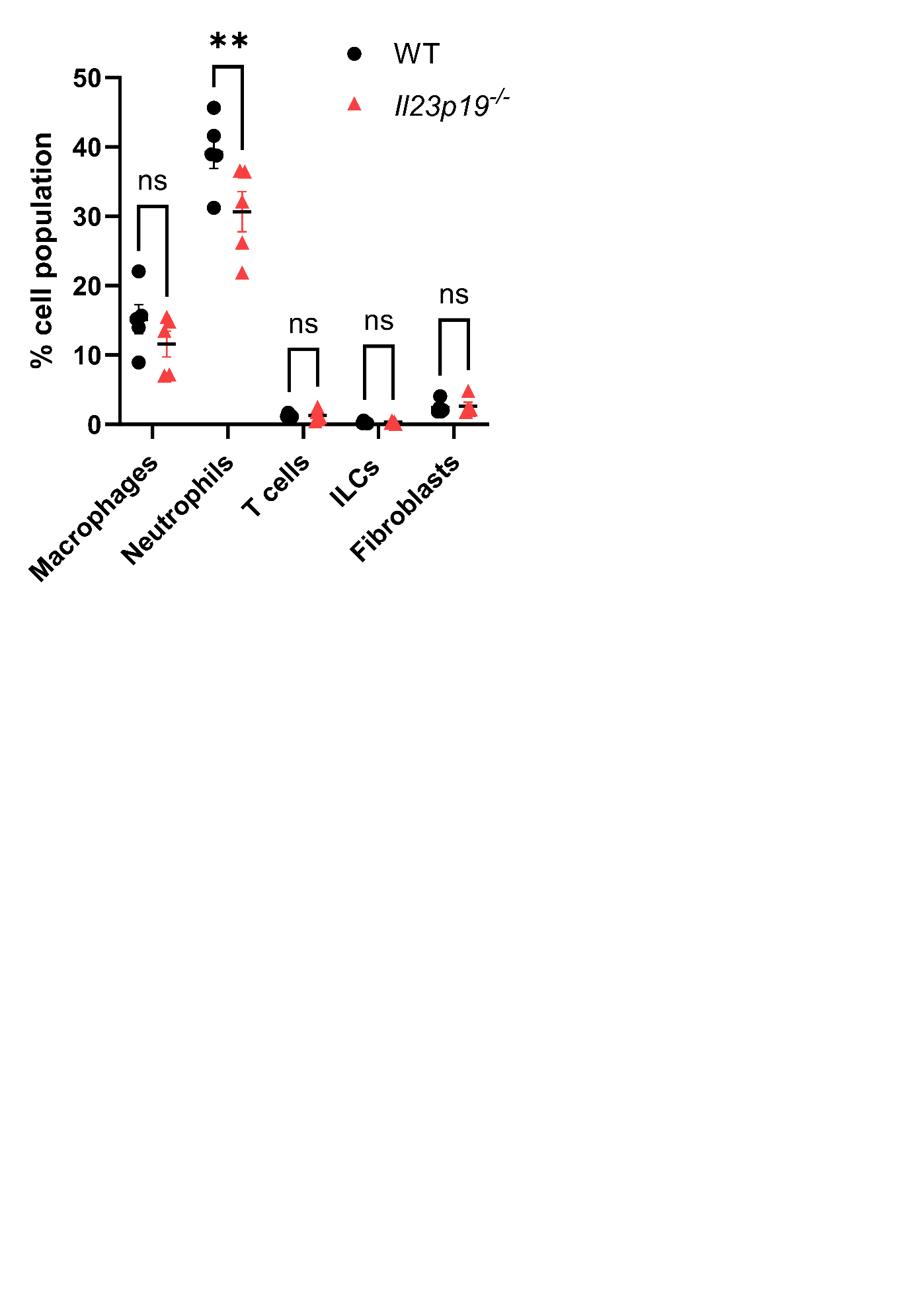
**

**Supplemental Fig. 1 Immune cell profile in ZIA joints.** Sorted ZIA synovial tissue cells from WT and *Il23p19^-/-^* cells from day 7 prior to scRNAseq analysis. The percentage of cell populations, namely macrophages, neutrophils, T cells, ILCs and fibroblasts, in ZIA joints was calculated and plotted.

Data are means ± SEM (n=5 mice/group); **p<0.01, WT vs. *Il23p19^-/-^* mice; ns, not significant.


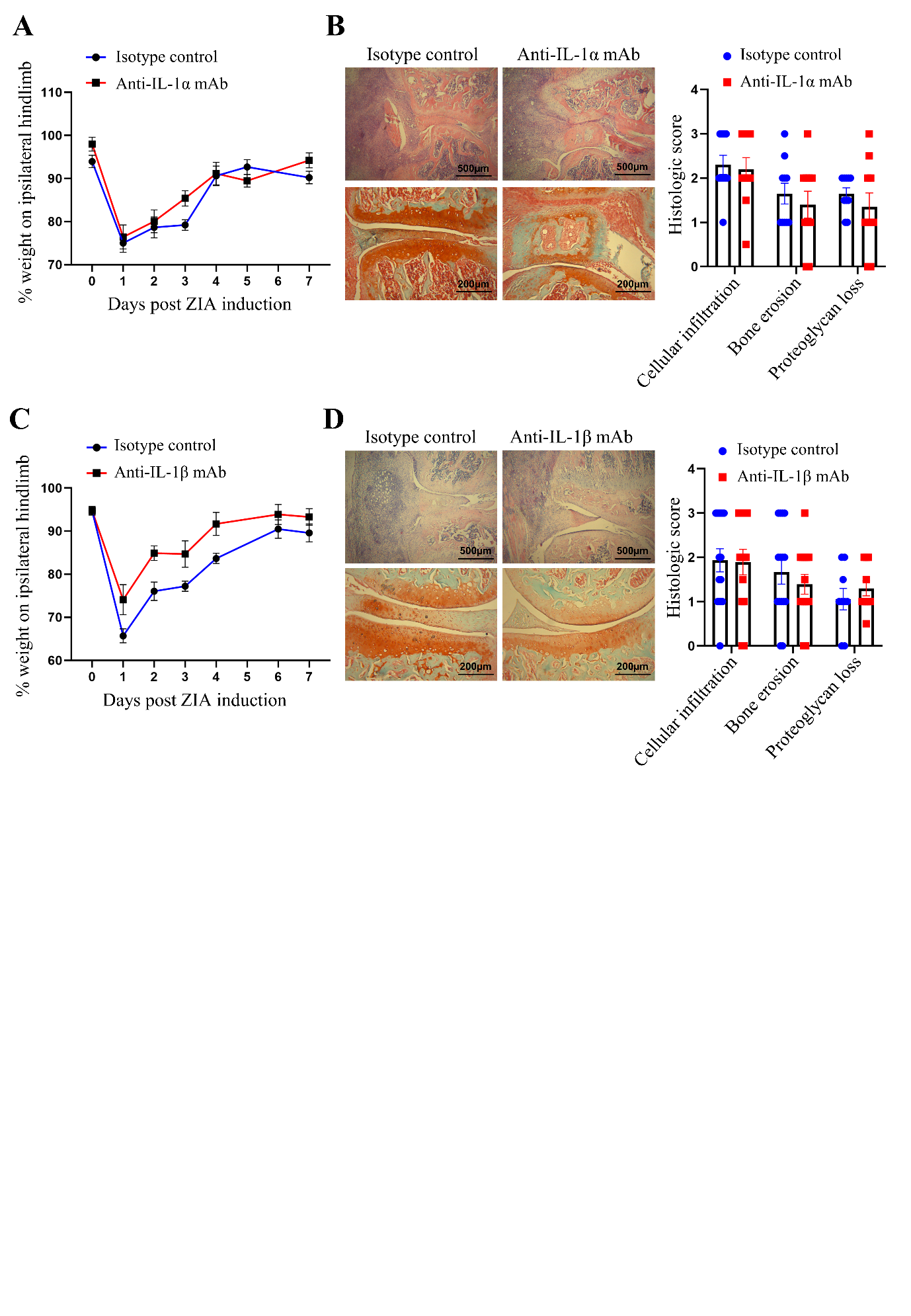


**Supplemental Fig. 2 IL-1α and IL-1β are not required for the development of ZIA pain and maximal disease.** (A and B) anti-IL-1α mAb (150µg) or (C and D) anti-IL-1β mAb (150µg) was administered prophylactically on day -1, 1 and 4 in WT mice with day 0 being the induction of the ZIA model. (A and C) Reduction in weight distribution (pain-like behaviour) over time. (B and D) Representative histologic pictures of knee joints (H&E, original magnification X40; Safranin O and Fast Green, original magnification X100) and quantification of arthritis at day 7.

Data are means ± SEM (n=10 mice/group).


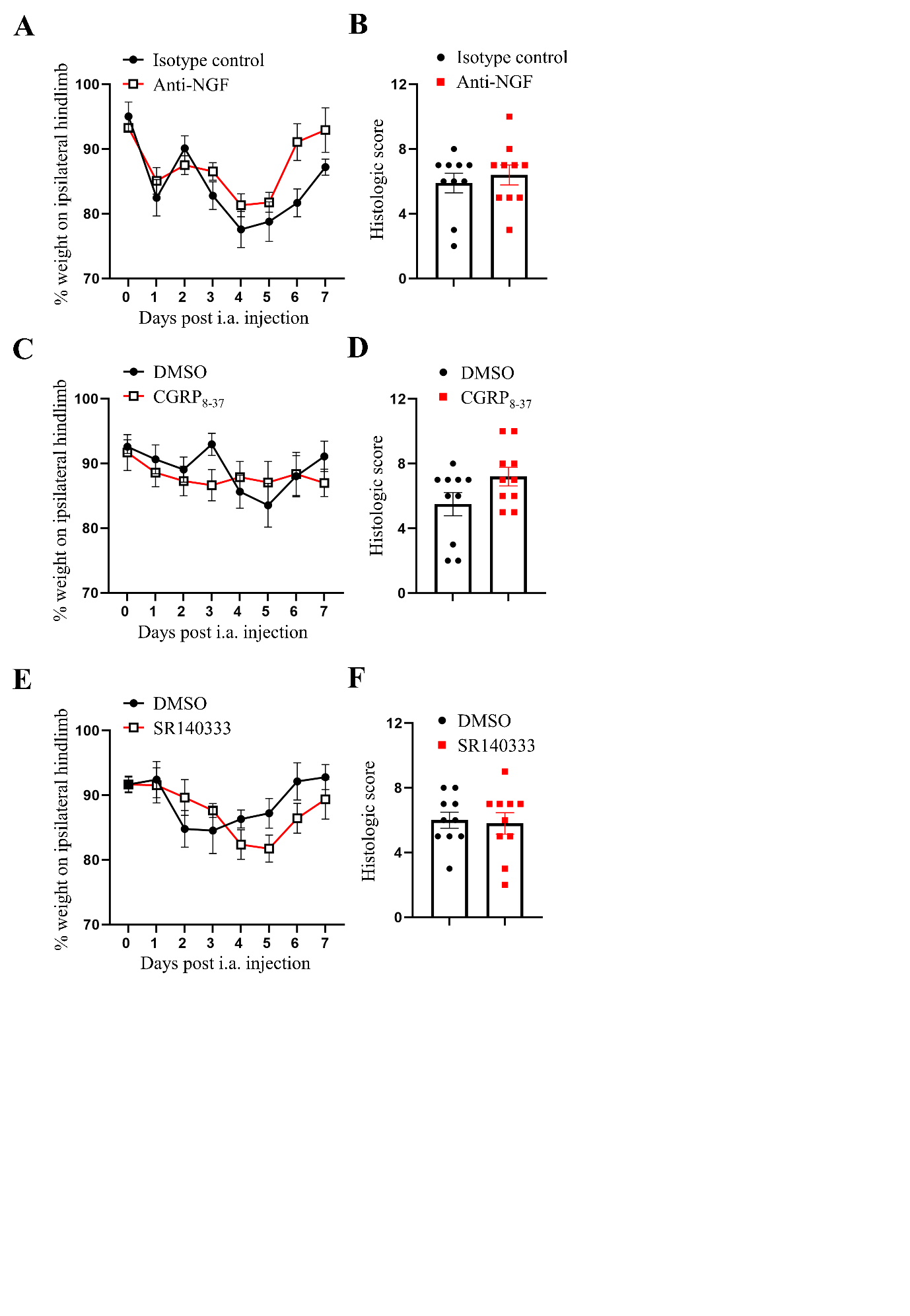


**Supplemental Fig. 3 IL-23-driven arthritic pain and disease do not require NGF, CGRP and substance P.** mBSA/IL-23 arthritis (i.a. mBSA [day 0] and s.c. IL-23 [5µg]) was induced in WT mice treated with (A and B) isotype control or anti-NGF mAb (150µg), (C and D) DMSO (control) or CGRP_8-37_ (1mg/kg) or (E and F) DMSO (control) or SR140333 (1mg/kg). The inhibitors were administered on days -1, 1 and 4. (A, C and E) Reduction in weight distribution (pain-like behaviour) over time. (B, D and F) Quantification of arthritis at day 7.

Data are means ± SEM (n=10 mice/group).

**REFERENCES**

1. Achuthan A, Cook AD, Lee MC, Saleh R, Khiew HW, Chang MW, et al. Granulocyte macrophage colony-stimulating factor induces CCL17 production via IRF4 to mediate inflammation. J Clin Invest. 2016;126(9):3453-66.

2. Lee KM, Zhang Z, Achuthan A, Fleetwood AJ, Smith JE, Hamilton JA, Cook AD. IL-23 in arthritic and inflammatory pain development in mice. Arthritis research & therapy. 2020;22(1):123.

3. Lee KM, Jarnicki A, Achuthan A, Fleetwood AJ, Anderson GP, Ellson C, et al. CCL17 in Inflammation and Pain. Journal of immunology. 2020;205(1):213-22.

4. Cook AD, Lee MC, Saleh R, Khiew HW, Christensen AD, Achuthan A, et al. TNF and granulocyte macrophage-colony stimulating factor interdependence mediates inflammation via CCL17. JCI Insight. 2018;3(6).

5. Lee MC, Saleh R, Achuthan A, Fleetwood AJ, Forster I, Hamilton JA, Cook AD. CCL17 blockade as a therapy for osteoarthritis pain and disease. Arthritis research & therapy. 2018;20(1):62.

6. Shin H, Prasad V, Lupancu T, Malik S, Achuthan A, Biondo M, et al. The GM-CSF/CCL17 pathway in obesity-associated osteoarthritic pain and disease in mice. Osteoarthritis Cartilage. 2023.

7. Lee MC, Lacey DC, Fleetwood AJ, Achuthan A, Hamilton JA, Cook AD. GM-CSF- and IRF4-Dependent Signaling Can Regulate Myeloid Cell Numbers and the Macrophage Phenotype during Inflammation. Journal of immunology. 2019;202(10):3033-40.
